# Endoscopy infection control strategy during the COVID-19 pandemic: experience from a tertiary cancer center in Brazil

**DOI:** 10.6061/clinics/2021/e2280

**Published:** 2021-03-01

**Authors:** Amanda A.M. Pombo, Luciano Lenz, Gustavo A. Paulo, Mônica A. Santos, Patricia K. Tamae, Alisson L.D.R. Santos, Daniel T. Rezende, Bruno Martins, Fabio S. Kawaguti, Caterina M.P.S. Pennachi, Carla C. Gusmon-Oliveira, Ricardo S. Uemura, Sebastian Geiger, Marcelo S. Lima, Elisa R. Baba, Viviane R. Figueiredo, Adriana Safatle-Ribeiro, Fauze Maluf-Filho, Ulysses Ribeiro-Júnior

**Affiliations:** Unidade de Endoscopia, Departamento de Gastroenterologia, Instituto do Cancer (ICESP), Hospital das Clinicas HCFMUSP, Faculdade de Medicina, Universidade de Sao Paulo, Sao Paulo, SP, BR

**Keywords:** COVID-19, Cancer, Endoscopy, Strategy

## Abstract

**OBJECTIVES:**

Strategic planning for coronavirus disease (COVID-19) care has dominated the agenda of medical services, which have been further restricted by the need for minimizing viral transmission. Risk is particularly relevant in relation to endoscopy procedures. This study aimed to describe a contingency plan for a tertiary academic cancer center, define a strategy to prioritize and postpone examinations, and evaluate the infection rate among healthcare workers (HCWs) in the endoscopy unit of the Cancer Institute of the State of São Paulo (ICESP).

**METHODS:**

We created a strategy to balance the risk of acute respiratory syndrome coronavirus 2 (SARS-CoV-2) infection and to mitigate the effects of postponing endoscopic procedures in oncological patients. A retrospective analysis of prospectively collected data on all endoscopies between March and June 2020 compared with those during the same period in 2019 was carried out. All HCWs were interviewed to obtain clinical data and SARS-CoV-2 test results.

**RESULTS:**

During the COVID-19 outbreak, there was a reduction of 55% in endoscopy cases in total. Colonoscopy was the most affected modality. The total infection rate among all HCWs was 38%. None of the senior digestive endoscopists had COVID-19. However, all bronchoscopists had been infected. One of three fellows had a serological diagnosis of COVID-19. Two-thirds of all nurses were infected, whereas half of all technicians were infected.

**CONCLUSIONS:**

In this pandemic scenario, all endoscopy services must prioritize the procedures that will be performed. It was possible to maintain some endoscopic procedures, including those meant to provide nutritional access, tissue diagnosis, and endoscopic resection. Personal protective equipment (PPE) seems effective in preventing transmission of COVID-19 from patients to digestive endoscopists. These measures can be useful in planning, even for pandemics in the future.

## INTRODUCTION

In mid-December 2019, a cluster of pneumonia cases associated with coronavirus disease (COVID-19) emerged in Wuhan, China, and rapidly spread to other areas globally (1). Subsequently, the World Health Organization declared that the pneumonia outbreak caused by the novel coronavirus was a public health emergency of international concern (pandemic) (2).

In Brazil, the first case of COVID-19 was confirmed by the Health Authorities on February 26, 2020, in São Paulo city (3). On March 19, 2020, the Medical Council of São Paulo State (CREMESP) recommended that surgeries, examinations, and elective consultations be postponed to prioritize care for patients diagnosed as having COVID-19 and, consequently, to safeguard limited resources such as Personal protective equipment (PPE) for care of suspected and confirmed cases of COVID-19. However, it was recommended that surgery, radiotherapy, chemotherapy, and other strategies for the treatment of cancer patients be continued (4).

Strategic planning for COVID-19 care has dominated the agenda of diagnostic services, which have been further restricted by the need for minimizing viral transmission to patients and healthcare workers (HCWs). The risk of acute respiratory syndrome coronavirus 2 (SARS-CoV-2) transmission was considered particularly relevant in relation to endoscopy, given the aerosol-generating nature of the endoscopic procedures (5). Several endoscopy societies and expert groups have published guidelines on the performance of endoscopy during the pandemic (Table 1) (6-8).

Since the outbreak of COVID-19, the diagnosis and treatment of patients with cancer have been subject to major challenges. Although oncologists are not frontline workers during this pandemic, special attention must be paid not only to patient protection but also to the protection of the entire healthcare team and their families. Simultaneously, action should be taken to minimize the impact of the pandemic on the management of oncological patients (9).

The risk of infection among HCWs is significant. According to one of the earliest records of infections in China, 29% of patients were HCWs. The characteristics of the virus and its transmission make endoscopy a potential route of infection (6). However, there is a significant risk associated with delaying endoscopic diagnosis, especially in time-critical conditions, such as gastrointestinal cancers. Whereas COVID-19 tragically accounted for over 200,000 deaths globally by the end of April 2020 (10,11), there were around 18 million cases of cancer worldwide and 10 million cases of cancer deaths in 2018, with colorectal and gastric cancer accounting for 17% of deaths (12). It has been conservatively estimated that delays in cancer diagnoses and treatment could be responsible for nearly 7,000 additional deaths in England and over 30,000 deaths in the USA (11). Therefore, the effects of the pandemic on the management of other diseases must be mitigated as much as possible to reduce the pandemic’s effect on overall mortality.

Thus, the aims of this study were as follows:

1. To describe the contingency plan of a tertiary academic cancer center as well as the adoption of several approaches suggested by endoscopy societies.
2. To define a reasonable strategy to prioritize and postpone examinations and to compare the number of procedures performed during the COVID-19 outbreak with that during the same period in 2019.
3. To evaluate the infection rate among HCWs in the endoscopy unit of the Cancer Institute of the State of São Paulo (ICESP-HCFMUSP).

## MATERIAL AND METHODS

### Workplace (the hospital)

ICESP is a part of the largest hospital complex in Brazil. On the basis of the strategy of State authorities, whenever possible, patients with a confirmed diagnosis of COVID-19 are transferred to Hospital das Clínicas. This quaternary hospital was exclusively transformed to receive patients with moderate and severe COVID-19 since March 30, 2020 (13). However, some patients who were already treated at our institute, and could not be transferred, continued their treatment in ICESP, even after the diagnosis of COVID-19.

The cases were categorized according to the criteria of the World Health Organization for COVID-19:

1. Suspected case
  - A patient with acute respiratory illness (fever and at least one sign/symptom of respiratory disease, *e.g.*, cough, shortness of breath) and a history of travel to or residence in a location reporting community transmission of COVID-19 during the 14 days before symptom onset.
    OR
  - A patient with any acute respiratory illness AND who had been in contact with a patient with a confirmed or probable diagnosis of COVID-19 (see definition of contact) in the last 14 days before symptom onset.
    OR
  - A patient with severe acute respiratory illness (fever and at least one sign/symptom of respiratory disease, *e.g.*, cough, shortness of breath, AND requiring hospitalization) and the absence of an alternative diagnosis that fully explains the clinical presentation.

2.Probable case

- A suspected case in which testing for SARS-CoV-2 is inconclusive.
  OR
- A suspected case in which testing could not be performed for some reason.

3. Confirmed case

- A person with laboratory-confirmed COVID-19, irrespective of clinical signs and symptoms

4. Definition of contact

- Contact is a person who fulfilled any of the following criteria 2 days before and 14 days after symptom onset in a probable or confirmed case:
  1. Face-to-face contact within 1 meter and for more than 15 minutes with a probable or confirmed case;
  2. Direct physical contact with a probable or confirmed case;
  3. Direct care of a patient with probable or confirmed COVID-19 without the use of appropriate PPE;
    OR
  4. Other situations as indicated by local risk assessments.

**Figure 1:**
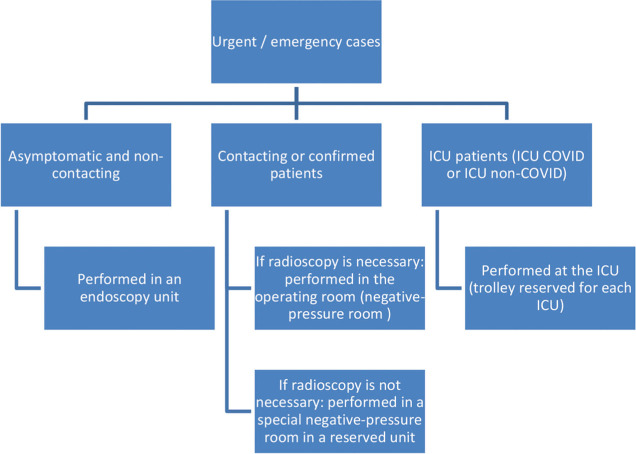
Inpatients.

In the intensive care unit (ICU), endoscopic procedures were performed at the bedside with a specific trolley. Patients in COVID-19 ICUs were already intubated or were intubated when performing the endoscopic procedures. During endoscopic examination in non-COVID-19 ICUs, orotracheal intubation was applied exclusively, according to the clinical condition. These recommendations were followed for both endoscopic digestive and respiratory examinations. However, for bronchoscopic procedures in intubated patients under mechanical ventilation in the ICU, the ventilation circuit was closed to prevent dispersion of viral droplets before bronchoscope insertion. For patients without gravity and hospitalized in regular wards, examinations were performed in a specific negative-pressure room located on the sixteenth floor in case of contact with a COVID-19 patient or a suspected or confirmed diagnosis of COVID-19. All other patients (without suspicion) were referred for examination at the endoscopy unit (Figure 1).

No outpatient examinations were performed for suspected or confirmed COVID-19 cases. All ambulatory examinations were performed in the endoscopy unit.

### Workplace (endoscopy unit)

Screening for respiratory symptoms such as running nose, cough, dyspnea, and fever and for contact with a COVID-19 patient was mandatory at the reception of our unit for access to the endoscopy unit. If the triage revealed positive results, the patient was directed to seek prompt medical attention at designated clinics, to quarantine, or to self-isolate, depending on the clinical scenario.

Our endoscopy unit consists of five rooms that lacked the facilities for negative-pressure creation. We have a designated area for admittance and preparation and another area for recovery. About 90% of bowel preparations for colonoscopy are performed in the unit, and the remaining 10%, at home.

After admission to the sector and clearance of the triage team, patients were shifted to the endoscopy rooms. Before entering the room, endoscopists followed all recommendations of the Institute with regard to PPE. Briefly, all involved professionals washed their hands with soap and water or alcohol-based hand sanitizer and wore a single-use, long-sleeved gown. Thereafter, an N95 (or FFP 2) mask was worn following standard recommendations. Protective goggles and a hairnet were worn, followed by a face shield. Finally, gloves were worn, covering the wrists.

On procedure completion, PPE removal was performed according to local and international recommendations. This was considered one of the most crucial steps of the examination and was carefully carried out to prevent self-contamination. Gloves were removed first, while still in the endoscopy room. Hand hygiene practices were followed using soap or alcohol-based hand rub. The gown was removed, and hand washing was repeated.

Outside the room, hand washing was repeated for the third time. Subsequently, the face shield, hairnet, goggles, and masks were removed in the described order. Touching the front of the face shield or goggles was to be avoided as they might have been contaminated by droplets or particles. Hand washing was repeated, the goggles were cleaned with alcohol or soap, and hand washing was repeated for the fifth time.

After each procedure, decontamination with chlorine or alcohol-based solutions was performed to clean the surface of the trolley, furniture, and floor of the endoscopic room.

There are no specific recommendations for the decontamination of endoscopes and accessories during the pandemic. Thus, we continued to follow the usual high-level disinfection protocols.

Our weekly scientific face-to-face seminar has been replaced by online meetings.

### Healthcare workers

Our endoscopic team consists of 19 physicians: 13 senior digestive endoscopists, 2 senior respiratory endoscopists, 3 advanced endoscopic fellows, and one second year fellow. However, the latter was temporarily away from our unit during the outbreak. As is common in Brazil, none of our doctors work exclusively at our hospital and have at least one other job. The nursing team comprises 18 technicians and six nurses.

In accordance with Brazilian law and as a preventive measure, all employees who were diagnosed as having COVID-19 or who were in a household with an infected individual were entitled to sick leave and were instructed to stay away from the hospital for 14 days to allow for home isolation. In addition, in accordance with hospital policy, all employees were subjected to serological SARS-CoV-2 testing. Thus, all HCWs in the endoscopy unit underwent serological SARS-CoV-2 testing at least once.

During the study, all HCWs were interviewed by one of the authors to obtain clinical data and SARS-CoV-2 test results.

### Procedures performed during the COVID-19 outbreak

A retrospective analysis of prospectively collected data on all respiratory and digestive endoscopy procedures between March 19 and June 19, 2020, compared with those during the same period in 2019 was carried out. This study was approved by the ethics committee (registration number: CapPesq/34797220.5.0000.0068).

Following the recommendations of CREMESP, we continued with all urgent digestive (*e.g.*, those for gastrointestinal bleeding, cholangitis, and foreign body retrieval) endoscopic procedures. We also continued to perform nutrition-associated procedures, including gastrostomy/jejunostomy, insertion of esophageal/duodenal stents, and nasoenteric tube insertion. We also continued to perform all procedures related to suspected or confirmed cases of pancreatobiliary neoplasia, particularly endoscopic ultrasound-fine needle aspiration (EUS-FNA) for its diagnosis and staging and endoscopic retrograde cholangiopancreatography (ERCP) to relieve obstructive jaundice.

All urgent respiratory procedures were continued (*e.g.*, hemoptysis and acute airway obstruction). We continued with diagnostic investigation by bronchoalveolar lavage and transbronchial and endobronchial biopsies. Endobronchial ultrasound-guided transbronchial needle aspiration (EBUS-TBNA) was also performed, especially for staging of lung cancer. Although bronchoscopy was not a part of the diagnosis and management protocols for COVID-19, this procedure was performed.

Examinations to investigate alarming symptoms (anemia, weight loss, dysphagia) as well as the others mentioned above were continued, as they were considered time-sensitive with a probable impact on the prognosis and survival of cancer patients. Endoscopic procedures to ablate or resect cancer (*e.g.*, endoscopic submucosal dissection) were also performed.

### Rescheduling examinations (Figure 2)

We postponed all non-essential endoscopic procedures between March 19 (date on which CREMESP recommended that elective procedures should be postponed) and June 19 (day with the highest number of new cases in São Paulo State, considering the peak of the outbreak).

For this, the following strategy was adopted, with the cooperation of the administrative team responsible for programing schedules:

1. Review of medical records with verification of the indication.
2. Telephone contact with the patient for screening respiratory symptoms, fever, and contact with a person diagnosed as having COVID-19.
3. If telephone contact was not possible and the patient visited the unit, steps 1 and 2 were carried out in person.
4. Individualization and establishing a balance between the risk of infection and benefit of the procedure.
5. Procedures considered nonurgent and non-time-sensitive were initially postponed to August 2020. Notably, examinations for investigating dyspeptic symptoms, screening colonoscopies, and post-polypectomy surveillance were included in this category.

## RESULTS

The study period was the most critical in terms of infection in our country. The number of new daily cases in Brazil increased from 193 on March 19, 2020, to 54,771 on June 19, 2020, (record day), an exponential growth of 147 times, turning São Paulo city into one of the most critical epicenters of the pandemic worldwide.

Between March 19 and June 19, 2020, there was a significant reduction in the number of endoscopic examinations. The total reduction was 55%. Colonoscopy was the most affected modality (−79%), followed by rectoscopy (−66%) and endoscopy (−46%). Enteroscopy, which has a limited volume and is mostly indicated for the diagnosis and possible treatment of bleeding, remained stable. There was no significant reduction in ERCP (−2.4%), and there was even an increase in the number of EUS procedures (+20%). There was also a significant decrease in the number of endoscopic respiratory procedures: by almost 40% for bronchoscopies and 50% compared to that in the same period in 2019 for EBUS (Table 2).

The total infection rate among all health professionals was 38% (16/42). None of the senior digestive endoscopists had COVID-19 (n=13). However, all bronchoscopists were infected (n=2). One of three fellows had a positive serological diagnosis for COVID-19. Two-thirds of all nurses were infected (4/6), whereas half of all technicians (9/18) were infected (Table 3). Of the 16 individuals diagnosed as having COVID-19, four were asymptomatic with only a serological diagnosis, and 12 had non-severe disease.

The PPE stock lasted for the entire outbreak, without shortage of any material.

## DISCUSSION

Since the declaration of COVID-19 as a global pandemic, healthcare systems worldwide have had to deal with the pandemic, and this situation is challenging for all clinicians (14). HCWs have a high risk of infection as they treat patients with COVID-19 (15). HCWs in endoscopy units are at a significant risk for respiratory diseases that can be transmitted via an airborne route, including aspiration of oral and fecal material via endoscopes (16). Further, late diagnosis and postponing therapeutic endoscopic procedures can have a major impact on the survival of cancer patients.

To the best of our knowledge, this is the first study that has described a rational strategy for performing endoscopic examinations, including respiratory and digestive diagnostic and therapeutic endoscopic examinations, in cancer patients in Brazil during the COVID-19 pandemic. Furthermore, in this study, the number of procedures during the outbreak was compared to that during the same period in 2019. In addition, this is the only study in which the infection rate of all symptomatic and asymptomatic employees was assessed by testing all endoscopists, technicians, and nurses in the endoscopy unit.

This study aimed to create a strategy to balance the risk for SARS-CoV-2 infection and mitigate the effects of postponing endoscopic procedures among oncological patients. The clinical phenotype and interactions of COVID-19 with pre-existing disease and systemic anticancer drug treatments have been poorly described. Disruption to normal oncological care due to the pandemic has been severe for several reasons. First, cancer clinicians and the rest of the cancer team are under unprecedented pressure. These pressures include increasing concern expressed by patients about their perceived vulnerability, canceled cancer operations, a substantial drive to practice telemedicine rather than conduct face-to-face consultations, and a high degree of absenteeism across the cancer team because of personal illness and self-isolation. Second, many oncologists are being redeployed to general or acute medicine roles to support in the treatment of COVID-19 patients who require intensive medical support and input (14). Third, some studies have concluded that cancer patients not only are more susceptible to contracting the virus than is the general population but also are at a risk of developing more severe sequelae (17,18). However, Lee et al. concluded that withholding effective cancer treatment from cancer patients during the pandemic is associated with the very real risk of an increase in cancer morbidity and mortality, perhaps much more so than that from COVID-19 itself (14).

Our strategy was based on some fundamental principles. The most crucial aspect was continuing with urgent procedures with an imminent risk of mortality. We also continued with all procedures associated with nutrition. Further, we continued performing procedures used for the diagnosis of neoplasms that have the worst prognosis (*e.g.*, pancreatic cancer). A total bilirubin level below 4-5 mg/dl is often required before starting chemotherapy, and endoscopic treatment is the method of choice for obstructive jaundice. Thus, we continued with ERCP and echoendoscopy as well. The number of ERCP procedures conducted between 2019 and 2020 was stable. However, there was an increase in EUS procedures, perhaps because of referral to our hospital for this procedure by other services that no longer performed the procedure. There was a general reduction in endoscopic examinations by 55%, with colonoscopy (79%) and rectoscopy (66%) showing more significant reductions. Although the risk of COVID-19 transmission during colonoscopy/rectoscopy because of the presence of the virus in the stool has been postulated, (19,20) this has not been well demonstrated and is currently considered unlikely (21). Our main concern was that these patients spent several hours in our sector during bowel preparation, in contact with several other patients. However, the evolution of colorectal cancer is very slow (22), and the overwhelming majority of postponed examinations were for patients who had already undergone at least one colonoscopy, further decreasing the probability of diagnosis of neoplasia; however, the possibility of diagnosis remained. Outpatient bowel preparation for colonoscopy was more practical for preventing COVID-19 transmission. The indications for performing colon preparation in the unit are multiple comorbidities, unfavorable clinical conditions, ostomized patients, or a high risk of malignant intestinal obstruction. These conditions are common at our center, which is why most bowel preparations were performed at the unit.

Because of the preventive measures, none of the senior doctors performing digestive endoscopy developed COVID-19. However, all bronchoscopists, 50% of the team of technicians, and 66.7% of the nurses were infected. Interestingly, although nurses were not present in the procedure room during the procedure at our hospital, the infection rate among nurses was high. Similarly, in a tertiary hospital in Wuhan, China, non-first-line young nurses were found to be more likely to be infected than first-line physicians aged 45 years or older (15). In the same study (15) and in an American study (23), no difference was observed between first-line and non-first-line HCWs. One limitation of this study was that we did not collect data from outsourced professionals (those responsible for cleaning and equipment disinfection) as they work in rotational shifts.

Another interesting fact is that no HCW related the contagion to the endoscopic procedure. Therefore, despite the initial fear and theoretical and rational risks of coronavirus transmission through endoscopic procedures, the risk seems small. According to a survey, of almost 1,000 Brazilian endoscopists, only 1% alleged being infected with SARS-CoV-2 because of exposure during endoscopic examinations (24). An Italian web-based survey reported a 4.3% positivity rate for COVID-19 among all employees in the endoscopy sector (21). Thus, in our study, the infection rate among endoscopists appears similar to that previously described, but the overall rate among all HCWs seems much higher than that in Italy. Some factors may explain this. First, the Italian study was an online poll survey (21), which may underestimate data. Second, in the study by Repici et al., the authors analyzed only symptomatic HCWs. In this study, all employees, both symptomatic and asymptomatic, were tested, and data were collected prospectively. Another aspect may be temporal. In Italy, data were collected for less than two months, whereas in our study data were collected for three months. In addition, the Repici et al. study did not mention whether the employees were only from the endoscopy sector or whether they occasionally transferred to other sectors, especially to the emergency room, such as the employees included in our study. Finally, the data may also reflect the epidemiological and geographic differences between São Paulo city and northern Italy.

The fact that the association between infection and endoscopic procedures has not been studied has its limitations. Self-reports have a considerable chance of being incorrect, especially in the scenario of a pandemic. However, it is also reasonable to consider that it is more likely to be infected during a shift in the ER, with dozens of infected patients; from prolonged contact with family members; or from social interaction with other employees (in the absence of PPE) rather than during a short-duration endoscopic procedure that involves the use of complete PPE. In addition, there were no reports in the 14-day period between the endoscopy of a patient known to be infected and the diagnosis of COVID-19 by any of the HCWs who participated in the procedure. Nevertheless, incubation periods can be longer, and transmission can also occur in patients without documented infection (25).

After this study, our goal will be to analyze the impact of delayed endoscopic examinations on the treatment and survival of cancer patients. An additional challenge we will face is with regard to minimizing the impact of rescheduling these endoscopic examinations.

## CONCLUSION

During this pandemic, endoscopy units should prioritize procedures to be performed. At our tertiary cancer center, it was possible to expand the variety of endoscopic procedures performed during the pandemic to include those for nutritional access, diagnosis of malignant neoplasia, and resection of malignant neoplasia, as all preventive measures to control infection spread had been taken. PPE seems effective in preventing the transmission of COVID-19 from patients to digestive endoscopists. However, special attention must be paid to avoid transmission in other sectors (if possible, by avoiding inter-sector transfer of employees) and transmission between HCWs. These measures can be useful in planning for pandemics in the future as well as for a possible second wave of COVID-19.

## AUTHOR CONTRIBUTIONS

Pombo AAM was responsible for the data curation, investigation, conceptualization, writing manuscript original draft, manuscript writing-review and editing, project administration, and study validation. Lenz L was responsible for the data curation, investigation, conceptualization, methodology, writing manuscript original draft, manuscript writing-review and editing, project administration, and study validation. Paulo GA was responsible for the data curation, investigation, conceptualization, writing manuscript original draft, and manuscript writing-review and editing. Santos MA, Tamae PK, Santos MA, Santos ALDR, Rezende DT, Martins B, Kawaguti FS, Pennachi CMPS, Gusmon-Oliveira CC, Uemura RS, Geiger S, Lima MS, Baba ER, and Figueiredo VR were responsible for the data curation, and investigation. Maluf-Filho F and Ribeiro-Júnior U were responsible for resources, conceptualization, formal analysis, methodology, visualization, manuscript writing-review and editing, project administration, and supervision. Safatle-Ribeiro A was responsible for the conceptualization, formal analysis, methodology, visualization, manuscript writing-review and editing, project administration, and supervision.

## Figures and Tables

**Figure 2:**
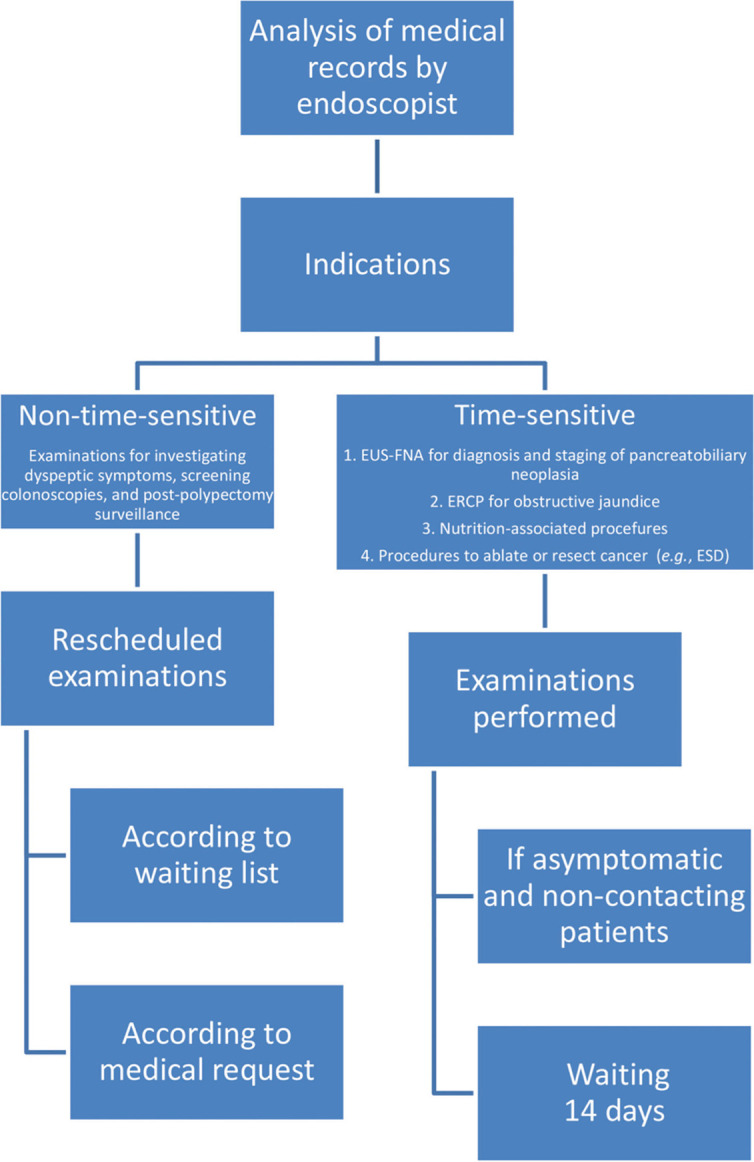
Out patients.

**Table 1:** Society *vs*. Recommendations.

SOCIETY	RECOMMENDATIONS
ASGE (American Society of Gastrointestinal Endoscopy)	In the pandemic area, the indications include management of upper gastrointestinal bleeding, acute cholangitis, foreign body, and obstructions. Care (initial diagnosis, biopsy, staging, palliation of biliary and luminal obstruction) of cancer patients may also be considered urgent. Reschedule nonurgent endoscopy services. This measure is aimed at reducing the risk of infection spread from asymptomatic patients, reducing the risk of cross-infection among patients, reducing the use of PPE*, and reducing unnecessary admissions to free up hospital resources.
ESGE (European Society of Gastrointestinal Endoscopy) and ESGENA (European Society of Gastroenterology and Endoscopy Nurses and Associates)	GI** endoscopy units should strongly consider temporarily postponing elective, nonurgent endoscopy procedures, according to availability of local human resources and local policies that may depend on regional/national pandemic rules/regulations
	1. The following list of GI endoscopy procedures should always be performed
	▪ Acute upper/lower GI bleeding with hemodynamic instability ▪ Capsule/enteroscopy for urgent/emergent bleeding ▪ Anemia with hemodynamic instability ▪ Foreign body in esophagus and/or high-risk foreign body in the stomach ▪ Obstructive jaundice ▪ Acute ascending cholangitis
	2. During the current COVID-19 pandemic, the following list of GI endoscopy procedures should be postponed with no need to reschedule before 12 weeks (low priority)
	▪ Surveillance for: Barrett’s esophagus without dysplasia or low-grade dysplasia or after endoscopic treatment, gastric atrophy/intestinal metaplasia, inflammatory bowel disease - primary sclerosing cholangitis ▪ Post-endoscopic resection (including immediate endoscopy after resection), surgical resection of cancer or post-polypectomy surveillance ▪ Diagnosis/surveillance of lynch syndrome and other hereditary syndromes ▪ Diagnosis of irritable bowel syndrome-like symptoms ▪ Diagnosis of reflux disease, dyspepsia (no alarm symptoms) ▪ Screening in high-risk patients for esophageal cancer, gastric cancer, colon cancer (primary screening endoscopy) or pancreatic cancer ▪ bariatric GI endoscopy procedures (e. g., intra-gastric balloons, endoscopic sleeve gastroplasty)
	3. Each of the following GI endoscopy procedures warrant a case-by-case evaluation according to medical necessity.
SOBED (Brazilian Society of Gastrointestinal Endoscopy)	All patients who are candidates for endoscopic procedures should be considered HIGH RISK.
	Thus, part of the endoscopic examinations considered ELECTIVE should be postponed until the outbreak is controlled, which will be duly communicated in subsequent updates to this recommendation.
	There are, however, some ELECTIVE examinations that can be considered ELIGIBLE for performance during the pandemic, considering the determinations of the local CRMs (National Medical Council), the local epidemiological situation, and the ability of the endoscopy service to fully comply with the biosafety determinations recommended by ANVISA (National Health Surveillance Agency).

* PPE: Personal protective equipment.

** GI - Gastrointestinal.

**Table 2a:** Digestive endoscopic procedures performed and variations between the two time periods.

	03/19/2019 - 06/19/2019	03/19/2020 - 06/19/2020	Variation
Endoscopy	1114	596	-46%
Rectoscopy	133	45	-66%
Colonoscopy	589	122	-79%
Enteroscopy	4	4	0%
ERCP*	41	40	-2.40%
EUS**	29	35	+20%
TOTAL	1910	842	-56%

* ERCP: Endoscopic retrograde cholangiopancreatography.

** EUS - Endoscopic ultrasound.

**Table 2b:** Respiratory endoscopic procedures performed and variations between the two time periods.

	03/19/2019 - 06/19/2019	03/19/2020 - 06/19/2020	Variation
Bronchoscopy	103	62	-39.80%
EBUS***	28	14	-50%
TOTAL	131	76	-42%

*** EBUS center Endobronchial ultrasound.

**Table 3:** Healthcare worker groups.

Group	TOTAL	Infected workers (%)
Digestive Endoscopists	13	0 (0)
Bronchoscopists	2	2 (100)
Fellows	3	1 (33.3)
Nurses	6	4 (66.7)
Technicians	18	9 (50.0)
TOTAL	42	16 (38.1)
